# Personal Protective Equipment Efficiency in Healthcare Emergencies: A Single-Center Experience

**DOI:** 10.7759/cureus.27823

**Published:** 2022-08-09

**Authors:** Harmeen Goraya, Nikhil Meena, Rajani Jagana

**Affiliations:** 1 Pulmonary and Critical Care, University of Arkansas for Medical Sciences, Little Rock, USA

**Keywords:** health care worker (hcw), coronavirus disease 2019, health care emergencies, acls, quality improvement, covid-19, cpr, aerosol, personal protective equipment (ppe)

## Abstract

Coronavirus disease 2019 (COVID-19) has dramatically shifted the healthcare landscape since 2020. Measures against it includes universal masking in the healthcare areas and the community, viral testing before aerosolizing procedures, and ambulatory elective surgical procedures. Some hospitals have had mandated viral testing policies even before admission to the hospital. Healthcare workers (HCWs) have been cautiously modifying all pertinent practices to avoid the transmission of the virus.

Personal protective equipment (PPE), including gowns, gloves, eye protection, and properly fitted N95 respirator or powered air-purifying respirators (PAPR) while treating the suspected and confirmed COVID-19 patients were made mandatory. Similarly, we changed our aerosol-generating procedures (AGPs) protocols based on available limited data. We amended our approach to in-hospital cardiopulmonary resuscitation (basic life support (BLS)/advanced cardiovascular life support (ACLS)), given the risk of aerosol generation and transmission during the process. This article shares our experience and outcomes of PPE use in healthcare emergencies at our tertiary care academic center.

## Introduction

Healthcare workers (HCWs) are at substantial risk for acute respiratory illness when exposed to microbial organisms during aerosol-generating procedures (AGPs). Many AGPs are performed routinely at our tertiary care center. An AGP is defined as any medical procedure that can induce the production of aerosols of diverse sizes [1]. Some of the commonly performed AGPs in healthcare settings include endotracheal intubation, mechanical ventilation, open suctioning of airways, bronchoscopy, non-invasive mechanical ventilation, manual bag and mask ventilation, and cardiopulmonary resuscitation [2]. While most AGPs are elective procedures, few are emergent, and a timely response is essential to save patients that eventually increase the risk of exposure to aerosols to HCW.

 In-hospital healthcare emergencies in our center are divided into two categories. Rapid response team (RRT) calls for life-threatening emergencies and code blue calls for cardiac and respiratory arrest, where advanced cardiac life support (ACLS) resuscitation measures are needed. A multidisciplinary team including physicians from anesthesiology, pulmonary and critical care medicine, internal medicine, respiratory therapists, and intensive care unit nurses respond to these calls. Hence, the total number of members is between 10 and 15 in the patient's room during this emergency. 

## Materials and methods

ACLS resuscitation measures include interventions such as high-quality chest compressions, bag and mask ventilation, electric or chemical cardioversions, endotracheal intubation, and the administration of life-saving medications. All the procedures except for medication administration are AGPs, and HCWs are at considerable risk of exposure to these aerosols. World Health Organization (WHO) provided infection prevention and control strategies amid the coronavirus disease 2019 (COVID-19) pandemic in March 2020 based on the experience of severe acute respiratory syndrome (SARS) and Middle East respiratory syndrome-corona virus (MERS-CoV) cases [3]. WHO recommended limiting the number of people in the room to the absolute minimum while performing AGPs. 

**Figure 1:**
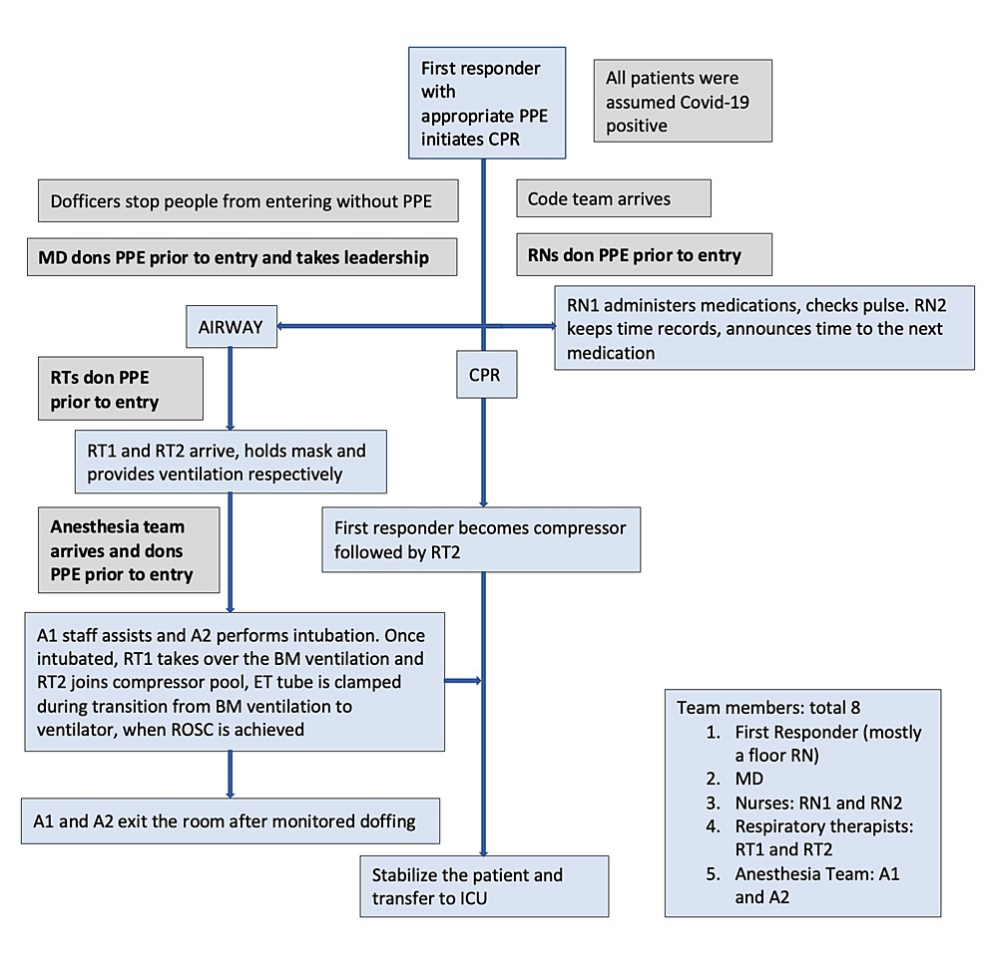
Modified ACLS response policy, March 2020 PPE: personal protective equipment; RT: respiratory therapist; CPR: cardiopulmonary resuscitation; COVID: coronavirus disease 2019; RN: registered nurse; MD: physician; A: anesthesia team; ROSC: return of spontaneous circulation; ACLS: advanced cardiac life support

In early March 2020, our institution, University of Arkansas for Medical Sciences (UAMS), Little Rock, Arkansas, United States, also revised the policies and protocols for the ACLS response team to minimize the risk of transmission of COVID-19 to HCWs (Figure 1). We provided stepwise guidance to put on (don) and take off (doff) personal protective equipment (PPE) in the protocol. All the HCWs who attended the code blue emergencies and rapid response calls in the hospital completed online sessions and supervised donning and doffing training. Donning included training for the technique and sequence of wearing masks, a powered air purifying respirator (PAPR), gown, and gloves. Doffing included the training for the technique and sequence of removing these PPE items. Team members were recommended to don and doff for all healthcare emergencies irrespective of the patient's COVID-19 infection status. The UAMS Medical Center, Little Rock, Arkansas, United States, enforced a maximum of eight HCWs during a code blue regardless of the patient's COVID-19 status. A staff member designated as the dofficer was charged with ensuring no one entered the room without PPEs. In real-time, all the donning and doffing techniques were monitored by the dofficer as well.

This retrospective observational study was exempted from IRB by the UAMS Institutional Review Board (Exemption IRB number: 274754). The reason for exemption was that we worked with deidentified HCW data provided by the infection prevention department, which was collected for internal quality improvement.

All the HCWs involved in these events were screened daily prior to entry to the hospital for covid-related symptoms via temperature measurement and an electronic questionnaire (COVID-19 screener survey) developed by the infection prevention department. The COVID-19 screener survey was part of an active tracer program; the program was in place to assure the efficacy of the changes made to policies to decrease occupational infection with COVID-19. All healthcare agencies/hospitals made policy changes based on previous pandemics and what we understood about the virus in question in that period. This information changed every few weeks and nothing was certain. We decided that we had to actively track every potential exposure at work, every possible COVID-19 infection in an HCW, and its potential relationship with an infection acquired at the medical center. The anonymized HCW data was available to the infection prevention and medical emergency response committee to develop interventions and adjustments to the policies in case of failure. Failure is HCWs being exposed to and then infected with COVID-19.

The COVID-19 screener survey included questions such as travel in and out of state, any direct contacts with confirmed covid cases with or without PPE, symptoms like sore throat, shortness of breath, cough, fever or chills in the last 24 hours, fever > 100.4F, new onset muscle ache not explained by exercise or activity, loss of taste or smell and any covid tests performed resulted positive or indeterminate. Symptoms developed 10 days after the event was deemed not related to that specific event. If HCWs developed symptoms, contact tracing was performed to identify the source of exposure.

Our purpose was to assess the COVID-19 transmission among the HCWs who attended these calls in the hospital since the policy change. All the RRT and code blue calls are reviewed every quarter at our center by the medical emergency response committee (MERC) to improve care quality and introduce new policies and education innovations. The recommendations by this committee have accomplished institution-wide improved patient care over the years, and we continued the process throughout the pandemic with virtual conferences. 

We extracted the patient data from the Viral Infection and Respiratory Illness Universal Study (VIRUS) registry, which was approved by the UAMS Institutional Review Board (IRB approval number: 260970). This registry is a part of the nationwide VIRUS registry, but our access was limited to our center-specific data. We also reviewed the data from our RRT and code blue calls from March to July, 2020.

## Results

Between March 2020 and July 2020, we had 87 code blues and 304 rapid response calls. Thirty-four patients had known COVID-19 infection during the event, and 47 patients tested positive for it after the event. A total of 673 HCWs were monitored for potential exposure to COVID-19 for 10 days during each code event. There were zero transmissions of COVID-19 infection to the HCWs. We were very encouraged by the efficacy of the PPE during healthcare emergencies.

Appropriate PPE was utilized for all the 391 emergencies, out of which 81 patients were confirmed positive for COVID-19 infection. PPE was used for healthcare emergencies even during the time of limited supply without any neglect. We studied the time to response and outcomes of all the 87 ACLS resuscitation calls separately, and the overall survival for patients who underwent ACLS resuscitation was 21%. The survival rate was similar to survival for resuscitated patients in the pre-pandemic years at our center. 

American Heart Association (AHA) provided updated ACLS guidelines in June 2020 [4]. The summary of adjustments to cardiopulmonary resuscitation (CPR) in suspected or confirmed COVID-19 patients as advised by AHA is depicted in Table 1. These guidelines are very similar to our institution's respiratory and ACLS protocols.

**Table 1:** Summary of adjustments to CPR by AHA PPE: personal protective equipment; CPR: cardiopulmonary resuscitation; AHA: American Heart Association; HEPA: high-efficiency particulate air

Reduce provider exposure
Don PPE before entering the room/scene
Limit personnel
Consider using mechanical CPR devices for adults and adolescents who meet height and weight criteria
Communicate COVID-19 status to any new providers
Prioritize oxygenation and ventilation strategies with lower aerosolization risk
Use a HEPA filter, if available, for all ventilation
Intubate early with a cuffed tube, if possible, and connect to mechanical ventilator, when able
Engage the intubator with highest chance of first-pass success
Pause chest compressions to intubate
Consider use of video laryngoscopy, if available
Before intubation, use a bag-mask device (or T-piece in neonates) with a HEPA filter and a tight seal
For adults, consider passive oxygenation with nonrebreathing face mask as alternative to bag-mask device for short duration
If intubation delayed, consider supraglottic airway
Minimize closed circuit disconnections
Consider resuscitation appropriateness
Address of goals of care
Adopt policies to guide determination, taking into account patient risk factors for survival

## Discussion

The COVID-19 pandemic was a new challenge that overhauled people's daily lives on numerous fronts. It is especially true for HCWs and, in some ways, even more unsettling. Healthcare emergency response was a pivotal aspect of the pandemic as it included AGPs. Overall chaos and the critical importance of efficiency, which generates a sense of urgency during these events, places the HCWs at a slightly higher risk than usual of contracting the disease. Hence, the main issue we addressed here is how to prevent such transmission during these emergent situations.

Our overall approach was to analyze the emergencies we had handled during the pandemic and our performance. Throughout the pandemic, we observed and drew inspiration from various national and international institutions, which had previously managed the disease. Accordingly, our intensive care unit staff worked tirelessly to develop new policies and adapted algorithms aligned with the national standard of care at that time [4]. Given the paucity of valid data amid abundant published literature, it was imperative to analyze and validate the effectiveness of the approach we had adopted. The most important result of our study was that there was zero transmission to HCWs exposed to these healthcare emergencies, which from our point of view, validates our reformed practice during these challenging times.

Overall, in-hospital mortality of COVID-19 patients who suffered from cardiac arrest has been reported to be high [5]. A single-center study from southwest Georgia showed 100% mortality of COVID-19 patients who suffered from in-hospital cardiac arrest. The southwest Georgian study group had a 90.5% African American population with multiple co-morbidities, which could explain this result, given the increased risk of hospitalizations and deaths in racial and ethnic minority groups [6]. In contrast to the above study, a multi-center study of the Cleveland Clinic system showed a survival rate of 22.4% in patients who suffered from in-hospital cardiac arrest from March to October, 2020 [7]. COVID-19 infection carries an elevated risk of mortality and morbidity and was ranked as the third leading cause of death in 2021, following heart disease and cancer [8]. Our study shows a similar survival rate as well.

WHO global surveillance for COVID-19, primarily in European and American regions, indicated that approximately 14% of COVID-19 cases are identified in HCWs [9]. A systematic review of COVID-19 infections and mortality of worldwide HCWs until May 2020 showed a 3.9% infection rate and 0.9% case fatality rate, with 37.2 deaths per 100 infections for HCWs aged 70 years or older [10]. COVID-19 infections and mortality among HCWs follow that of the general population. HCWs working in high-risk departments were found to have an increased risk of acquiring COVID-19 infection [11]. Standardized care with appropriate infection prevention and control is necessary to save patients and health care workers, and our current study stresses the same.

Other studies have attempted to identify the environmental factors affecting transmission and to see the effectiveness of non-pharmacological interventions in decreasing the transmission rate in the community, which indicates the importance of addressing this issue [12,13]. Another factor that places HCWs at a higher risk of contracting the COVID-19 disease is HCW to HCW transmission; therefore, daily screenings of HCWs have been arranged in institutions, including ours, to reduce in-hospital transmission [14].

The major limitations of the current study are the retrospective observational design and the small sample size. Our primary focus was to look at the transmission rate and not patient outcomes; as the result was the absence of transmission in this sample size, the effect still remains large and makes our study a positive one. We can argue the utility of performing a bigger prospective cohort study where we can also look at patient population variables and outcome measures. There is a need for future studies to determine other ways to reduce transmission to HCWs other than PPEs.

## Conclusions

There are many misconceptions during the pandemic, from the utility of facemask use to COVID-19 vaccine efficacy. Our observation reinforces that the appropriate use of PPE, even in in-hospital emergencies, is remarkably effective in providing the staff with protection without a notable change in the workflow. Healthcare systems should ensure adequate availability of PPE and develop strategies to avoid transmission of COVID-19 infections to HCWs. Our observation is mounting support for the fact that PPEs save lives. These strategies and protocols also guide the healthcare community in facing future infectious challenges. They help protect the HCWs while providing the best available care to the general population.
